# Proof of principle concept for the analysis and functional prediction of rare genetic variants in the *CYP2C19* and *CYP2D6* genes

**DOI:** 10.1186/s40246-025-00765-2

**Published:** 2025-05-28

**Authors:** Inger Johansson, Yuchen Lu, Yitian Zhou, Kristi Krebs, Martina Akcan, Lili Milani, Magnus Ingelman-Sundberg

**Affiliations:** 1 Department of Physiology and Pharmacology, Karolinska Institutet, 171 77 Stockholm, Sweden; 2 Center for Molecular Medicine, Karolinska Institutet and Karolinska University Hospital, Stockholm, Sweden; 3 Estonian Genome Centre, Institute of Genomics, University of Tartu, Riia 23B, 51010 Tartu, Estonia

**Keywords:** Pharmacogenomics, Clopidogrel, Omeprazole, Antidepressants, Antipsychotics

## Abstract

**Background:**

Variations in pharmacogenes that regulate drug absorption, distribution, metabolism, and excretion (ADME) contribute to approximately 20–30% of interindividual differences in drug response. While many common variants are successfully utilized in clinical settings to predict individual drug responses, a significant portion of the genetic basis underlying this variability remains unidentified. This includes rare variants, which are estimated to account for 4–6% of drug response variability.

**Results:**

To comprehensively elucidate the functional consequences and molecular mechanisms of rare variants, we conducted in vitro enzyme expression studies combined with in silico structure–function analyses. We selected 11 rare variants in the *CYP2C19* and *CYP2D6* genes identified among participants within the Estonian Biobank. Variant cDNAs were heterologously expressed in HEK-293 cells, and detailed enzyme activity analyses were performed. The experimental results were further validated against average scores from five optimized in silico prediction models: LRT, Mutation Assessor, PROVEAN, VEST3, and CADD. To explore structure–activity relationships, we performed in silico docking of substrates into available 3D enzyme structures. Our findings reveal that most of the rare genetic variants caused significant functional alterations, including: (i) Likely impairments in substrate transport to the active site due to narrowing of access channels; (ii) Changes in catalytic rates; and (iii) Potential effects on substrate extrusion rates from the active site. The in silico prediction tools accurately anticipated the functional impact of 6 out of the 11 variants (54%).

**Conclusions:**

Evaluating the functionality of rare variants will become increasingly essential as rapid and cost-effective whole-genome sequencing technologies continue to advance. Our results highlight the need for further refinement of in silico prediction models, particularly those leveraging 3D crystal enzyme structures, to enhance the accuracy of functional predictions for rare genetic variants.

**Supplementary Information:**

The online version contains supplementary material available at 10.1186/s40246-025-00765-2.

## Introduction

The effectiveness of a particular drug treatment can be influenced by various intrinsic and extrinsic factors. A major problem of drug treatment is adverse drug reactions; in the US in 2022, more than 1.25 million serious adverse events and nearly 175,000 deaths were reported [1]. Genetic variation in pharmacogenes involved in drug absorption, distribution, metabolism, and excretion (ADME) account for approximately 20–30% of altered drug response [2, 3]. Among these pharmacogenetic variants, polymorphic genes encoding cytochrome P450 enzymes play a crucial role in determining interindividual variability in drug response [3]. Indeed, FDA has identified CYP2C19 and CYP2D6 genetic polymorphism as the most important pharmacogenomic factors for predicting drug efficacy and metabolism of psychoactive drugs [4]. CYP2C19 and CYP2D6 are key enzymes in the metabolism of several antidepressants and antipsychotics, including escitalopram, sertraline, and fluoxetine (CYP2C19 substrates), as well as risperidone, aripiprazole, venlafaxine, and vortioxetine (CYP2D6 substrates). Several studies have highlighted the importance of these enzymes in psychiatry [5–7].

Numerous allelic variants of the *CYP2C19* and *CYP2D6* genes have been identified [8]. Based on their genotype, patients can be classified as ultra-rapid metabolisers (UMs), normal metabolisers (NMs), intermediate metabolisers (IMs) or poor metabolisers (PMs). As a result of faster and cheaper genome sequencing, more and more rare functional genetic variants are being identified. Indeed among pharmacologically relevant CYP genes, rare variants have been estimated to explain from 2.8% of the genetically encoded functional variability in *CYP2B6* up to 17.5% for *CYP3A4*, and are estimated to account for 4.4% and 6.3% of the total genetic variability in *CYP2C19* and *CYP2D6* function, respectively [3]. Indeed, it is of importance to identify the function of such genetic variants in order to customise drug treatment. Since most of the rare CYP variants are causing amino acid substitutions, characterization of the functionality of these is best using heterologous expression systems. There are several functional prediction models for metabolic phenotypes based on genetic variations that generally predict the resulting phenotype with an accuracy of 50–60% [9]. The level of predictability based on pharmacogenomics is expected to evolve rapidly with advances in whole-genome analysis through long-read sequencing techniques that enable the identification of thousands of relevant rare genetic variants and their phase in relation to other previously known genetic variants [3].

In this study, we selected rare genetic variants detected in *CYP2C19* and *CYP2D6* among participants from the Estonian Biobank to thoroughly determine the functional consequences and molecular basis for altering their functionality using in vitro enzyme variant expression studies in combination with in silico structure–function analyses. We found that the majority of the genetic variants caused significant functional changes that apparently altered substrate transport to the active site, catalysis rate or likely the extrusion rate from the active site. We conclude that further development of in silico prediction models is important to capture the functionality of rare genetic variants, where substrate-enzyme interactions based on substrate/metabolite- enzyme docking models would also be important.

## Material and methods

### Variant identification within the Estonian Biobank sequencing data

Novel putative missense variants were identified in the whole genome (n = 3000) and whole exome (n = 2500) sequencing data of Estonian Biobank participants. Sequencing data processing, variant calling, and filtering are described elsewhere [10–12]. Variants were annotated using Variant Effect Predictor [13]. Altogether, we identified 15 novel putatively deleterious variants within the *CYP2C19* and *CYP2D6* genes (Table 1), including 11 missense variants (7 in the *CYP2C19* gene and 4 in the *CYP2D6* gene) that are predicted as deleterious based on the functionality prediction framework optimized for pharmacogenes [14], as well as four frameshift variants identified in the *CYP2D6* gene. Due to the clear truncating effect of the four frameshift variants that causes loss-of-function proteins, they were not further investigated in this study.

**Table 1:** Genetic variants in the *CYP2C19* and *CYP2D6* genes identified in the Estonian Biobank. Positions are according to GRCh37

rs-number	Position	Ref.	Alt.	MAF % (EstBB)	Amino acid exchange
*CYP2C19*					
rs1466428833	Chr10:96,535,156	T	G	0.02	F114C
rs1361528097	Chr10:96,540,264	T	G	0.02	C164G
rs140278421	Chr10:96,540,331	G	A	0.04	R186H
rs148247410	Chr10:96,541,688	C	G	0.02	H251Q
rs1431015009	Chr10:96,580,257	A	T	0.02	K275M
rs1393133490	Chr10:96,580,323	C	T	0.02	A297V
Not available	Chr10:96,602,648	T	C	0.02	M339T
*CYP2D6*					
rs1230912765	Chr22:42,525,143	G	A	0.02	R133C
rs1058171	Chr22:42,523,928	C	T	0.02	D301N
Not available	Chr22:42,523,916	C	T	0.02	A305T
Not available	Chr22:42,523,889	G	T	0.02	L314M
rs28371733	Chr22:42,522,916	CG	C	0.02	E418NfsTer11
rs757396767	Chr22:42,522,982	TGA	T	0.10	L395HfsTer11
rs730882170	Chr22:42,523,847	GCACATCCGG ATGTAGGATC	G	0.02	M321IfsTer12
rs750439337	Chr22:42,524,820	T	TC	0.92	E211GfsTer43

### Prediction of the functional activities of new CYP2C19 and CYP2D6 variants

The functional effects of the previously uncharacterised missense variants of *CYP2C19* and *CYP2D6* identified in the EstBB [15] (Table 1) were assessed using the ADME-optimised framework, a method that was previously established tailored for predicting functional consequences of pharmacogenetic variants (Ref 14). This framework was trained on 337 functionally well-characterized variants from 43 ADME genes and integrates five orthogonal variant prediction models (LRT, MutationAssessor, PROVEAN, VEST3, and CADD) to achieve superior prediction sensitivity and specificity compared to 18 other commonly used variant prediction tools. The resulting prediction scores can be further translated into enzyme activity levels relative to those of wild-type enzymes [14].

### Expression plasmids

The expression plasmid pCMV3 containing CYP2C19*1 cDNA, respectively, was obtained from Sino Biological Europe GmBH (Eschborn, Germany) and the expression plasmid pCMV4 containing *CYP2D6.1* was available inhouse. Mutants of *CYP2C19* and *CYP2D6* cDNA were produced using the QuikChange Lightning Site-Directed Mutagenesis Kit (Agilent, Santa Clara, CA, USA) according to the manufacturer’s protocol. The sequences of the mutagenesis primers are presented in Supplementary Table 1. All cDNAs were verified by DNA sequencing at the KI Gene core facility, Karolinska Institutet. Plasmids were isolated using the Qiagen Plasmid Plus Midi kit (Hilden, Germany).

### Transient expression of CYP2C19 and CYP2D6 variants

The CYP2C19 and CYP2D6 enzyme variants were expressed in HEK293 cells grown in DMEM containing 4.5 g/L glucose, 10% fetal bovine serum, and penicillin–streptomycin (100 U/mL and 100 μg/mL) to 70–80% confluence in 6-well plates. The pCMV expression plasmids containing the *CYP2C19* and *CYP2D6* cDNA variants were transfected using Lipofectamine™ 3000 Transfection Reagent (Invitrogen, ThermoFisher Scientific, Waltham, MA, USA) according to the manufacturer’s protocol. Cells were harvested after 48 h of incubation, and cell pellets were stored at − 80 °C. Cell pellets were resuspended in 100 mM sodium phosphate buffer, pH 7.4, sonicated 20 times for 1 s each, and centrifuged at 800 ×*g* for 10 min. The supernatants were aliquoted and stored at − 80 °C.

Protein determinations were performed using *DC* Protein Assay Reagents (BioRad) and SpectraMax iD3 spectrophotometer (Molecular Devices LCC.). Expression levels were verified with SDS-WB analysis with CYP2C19 antibodies from Sigma, Merck (HPA015066) and CYP2D6 antibodies from Daiichi, Tokyo, Japan. Similar expression levels were obtained between allelic variants and between experiments.

### Omeprazole hydroxylation

CYP2C19 catalytic activity was determined by analysing the rate of 5-hydroxylation of omeprazole. Incubations were performed at 37 °C with 800 × g supernatant corresponding to 300 μg protein, 35 μM omeprazole, and 1 mM NADPH in a total volume of 300 μL 100 mM sodium phosphate buffer, pH 7.4. After 30 min, the incubations were transferred to ice and processed as described by Baldwin et al. [16], using lansoprazole as the internal standard. Data are presented as average ± SD and is from incubations with cell lysates from three independent expressions in HEK293 cells.

### Bufuralol hydroxylation

CYP2D6 catalytic activity was determined by analysing the rate of bufuralol hydroxylation. Incubations were performed with 800 ×*g* supernatant corresponding to 25–125 μg of protein, 0.1 M sodium phosphate buffer, 50 μM bufuralol (racemate), and 1 mM NADPH in a total volume of 150 μL. Reactions were incubated for 2–5 h and terminated by the addition of 14 μL of 70% perchloric acid. After centrifugation, the supernatant was analysed by HPLC as described by Kronbach et al. [17]. Data are presented as average ± SD and is from incubations with cell lysates from three independent expressions in HEK293 cells.

### Protein and ligand preparation

The crystal structure of human CYP2C19 (PDB ID: 4GQS) and CYP2D6 (PDB ID: 3TBG) were obtained from the Protein Data Bank and visualized using UCSF Chimera [18]. All co-crystallized ligands, except the heme molecule, along with water molecules and chains B, C, and D, were removed from the two crystal structures and hydrogen atoms were added to the protein complex. Variant protein structures were predicted using the Rotamer function in UCSF Chimera or constructed using AlphaFold 2 Colab [19]. The 3D structure of the ligand, omeprazole and bufuralol, was obtained from PubChem (CID: 4594 and 71,733).

### Docking

The 3D structure of omeprazole was docked into the CYP2C19.1 structure using AutoDock Vina 1.1.2 in UCSF Chimera. Omeprazole was docked against the entire CYP2C19 protein. The docking area and dimensions were defined as center_x: -80.8682, center_y: 20.1386, center_z: -44.8835, size_x: 65.1899, size_y: 72.6923, and size_z: 67.5182. The default parameters were used for the receptor, ligand, and advanced options. Nine poses with different RMSD values were generated, and the pose with a score of -8.7 and an RMSD of 0.0 was retained for further analysis. Bufuralol was docked against the CYP2D6 active site [20]. The docking area and dimensions were defined as center_x: 6.16969, center_y: 24.2858, center_z: -7.48951, size_x: 20.2914, size_y: 30.079, and size_z: 27.1963. The default parameters were used for the receptor, ligand, and advanced options. Ten poses with different RMSD values were generated, and the pose with a score of -8.0 and an RMSD of 0.0 was retained for further analysis.

### Contacts and clashes analysis

Hydrogen bonds, contacts, and clashes were identified using the built-in functions in UCSF Chimera [18]. Clashes were defined as unfavorable interactions where atoms exhibited van der Waals overlaps of 0.6 Å or greater. If hydrogen bonds were present, 0.4 Å was subtracted from the overlap. Contacts were defined as all direct interactions, including unfavorable clashes, with van der Waals overlaps of − 0.4 Å or greater. The relax constraints of hydrogen bonds were 0.4 Å and 20 degrees. The atomic distances at the sites of clashes occur were quantified in two ways. If one clash is identified between an atom in the mutated residue and an atom in the side chain of the surrounding amino acid residue (or the ligand), distance between the two atoms was calculated directly. If one clash is identified between an atom in the mutated residue and an atom in the backbone of the surrounding amino acid residue, then the distance between the atom in the mutated residue and the alpha carbon in the surrounding amino acid residue is quantified as a reference. 

### Access channels analysis

The access and egress channels of CYP2C19 with the heme molecule were calculated using MOLEOnline [21]. Default settings were used, with the origin radius set to 5 and the surface cover radius set to 10. The channels of CYP2C19 were defined based on the methodology of Cojocaru, Winn, and Wade [22], while the channels of CYP2D6 were modelled based on the description of Márquez et al. [23].

## Results

### ADME-optimized algorithm prediction for activity of the CYP2C19 and CYP2D6 variants

To assess the potential functional impact of the rare genetic variants in CYP2C19 and CYP2D6, we used the ADME-optimised algorithm developed by Zhou et al., which is based on the LRT, Mutation Assessor, PROVEAN, VEST3 and CADD models [14]. As shown in Table 2 and Supplementary Table 2, all variants resulted in ADME scores above 0.5, indicating deleterious effects and impaired enzyme function. The ADME scores were converted into percentages representing the activity of the protein variant compared to the reference enzymes CYP2C19.1 and CYP2D6.1 (Table 2), based on a previously determined correlation between ADME scores and the experimentally determined functional in vitro activity of protein variants [14]. The CYP2C19 A297V variant had the highest ADME score of 1, which corresponds to an activity of 0% compared to CYP2C19.1. The R186H and M339T variants had the lowest ADME scores of 0.6, which corresponds to about 20–30% of the activity of CYP2C19.1. Other protein variants had ADME values of 0.8, which corresponds to 0–10% of the activity of CYP2C19.1.

**Table 2:** Comparison of the results predicted by the ADME optimized algorithm for the CYP2C19 and CYP2D6 variants, and the results obtained in the in vitro experiment

Amino acid exchange	ADME score^1^	ADME predicted activity compared to CYP2C19.1/ CYP2D6.1 (%)	ADME prediction*	Average activity (%) compared to CYP2C19.1/ CYP2D6.1 ± SD
CYP2C19				
F114C	0.8	0–10%	D	18.8 ± 10.7
C164G	0.8	0–10%	D	26.3 ± 9.5
R186H	0.6	20–30%	D	20.7 ± 6.3
H251Q	0.8	0–10%	D	3.7 ± 5.8
K275M	0.8	0–10%	D	36.6 ± 5.8
A297V	1	0	D	2.9 ± 4.4
M339T	0.6	20–30%	D	70.6 ± 35.1
CYP2D6				
R133C	1	0	D	4.6 ± 2.6
D301N	0.6	20–30%	D	0.9 ± 0.9
A305T	1	0	D	9.6 ± 3.3
L314M	0.8	0–10%	D	58.7 ± 24.0

^*^D means deleterious

Predictions for all CYP2D6 enzyme variants also showed ADME values ≥ 0.6, indicating impaired CYP2D6 function. Two of the variants, R133C and A305T, were predicted to lack catalytic activity, with ADME scores of 1. The D301N and L314M variants had scores of 0.6 and 0.8, respectively, corresponding to 0–30% of normal activity.

### Relative catalytic activity determined for CYP2C19 and CYP2D6 variants.

**Fig. 1:**
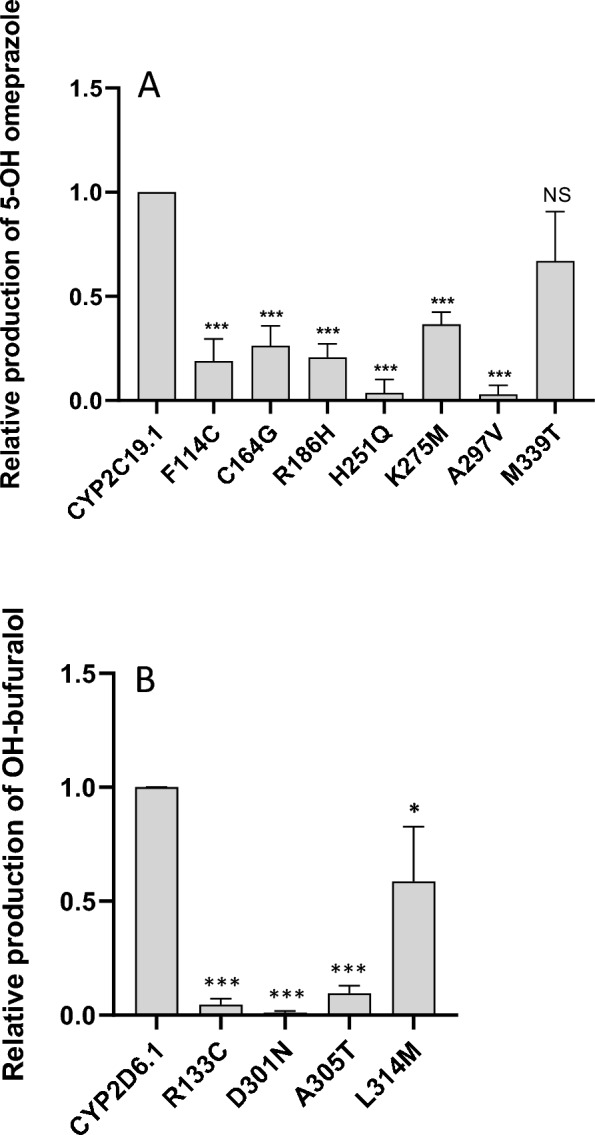
Relative catalytic activity by different CYP2C19 (**A**, omeprazole hydroxylation) and CYP2D6 (**B**, bufuralol hydroxylation) variants compared to CYP2C19.1 and CYP2D6.1, respectively. Data are presented as average ± SD and is from incubations with cell lysates from three independent expressions in HEK293 cells

To assess the catalytic activity of the seven CYP2C19 variants in comparison to CYP2C19.1, we measured the production of 5-hydroxy-omeprazole from omeprazole. As shown in Fig. 1A, six of the variants exhibited significantly lower catalytic activity compared to the CYP2C19.1 enzyme. In particular, the H251Q and A297V variants showed the lowest catalytic activity, while the M339T variant appeared to have normal catalytic activity. The remaining CYP2C19 variants showed low-to-moderate metabolizing capabilities for omeprazole compared to CYP2C19.1.

The catalytic activities of the four CYP2D6 variants compared to CYP2D6.1 were determined using a bufuralol hydroxylation assay (see Fig. 1B). Two of the variants, R133C and A305T, showed very low activity, while D301N showed almost no catalytic activity. The CYP2D6 variant L314M showed slightly lower activity than CYP2D6.1.

### Comparison of in vitro metabolism with prediction by algorithms

The omeprazole hydroxylation capacities of the seven CYP2C19 variants were compared to the predictions made by the ADME algorithm (Table 2). Notably, the H251Q and A297V variants showed minimal discrepancies between the predicted and observed catalytic activities, suggesting that the algorithm effectively captured the functional impacts of these two variants. This observation is consistent with the broader trend, where the algorithm demonstrated a 93% accuracy rate in predicting proteins with 0–10% functionality compared to the reference enzyme variant [14].

For other variants with 20–40% catalytic activity relative to CYP2C19.1, such as F114C, C164G, and K275M, the algorithm's predictive accuracy was notably less reliable. Additionally, the M339T variant, which exhibited near-normal function, was incorrectly predicted by the algorithm to have a deleterious impact. These discrepancies highlight the algorithm's limitations in accurately predicting proteins with intermediate activity levels (20–60% and 70–80% of the reference CYP2C19.1 activity), as reflected by a decline in the ADME score for these variants [14].

For the CYP2D6 variants, the predictions for R133C and A305T aligned closely with the catalytic activities observed in the bufuralol hydroxylation assay (Table 2). The R133C variant was predicted to lack enzymatic activity, and the in vitro assay confirmed this with only 5% of the activity of CYP2D6.1. Similarly, the A305T variant was predicted to be inactive and exhibited 10% of the catalytic activity of the reference enzyme. However, the algorithm was less accurate for two other variants. The D301N variant was predicted to retain 20–30% of CYP2D6.1 activity, but the assay revealed an almost complete loss of function during bufuralol incubation. Conversely, the L314M variant was predicted to have 0–10% activity but demonstrated nearly 60% of the reference CYP2D6.1 activity in the experimental results.

### Structural effects of CYP2C19 and CYP2D6 genetic variants

**Fig. 2:**
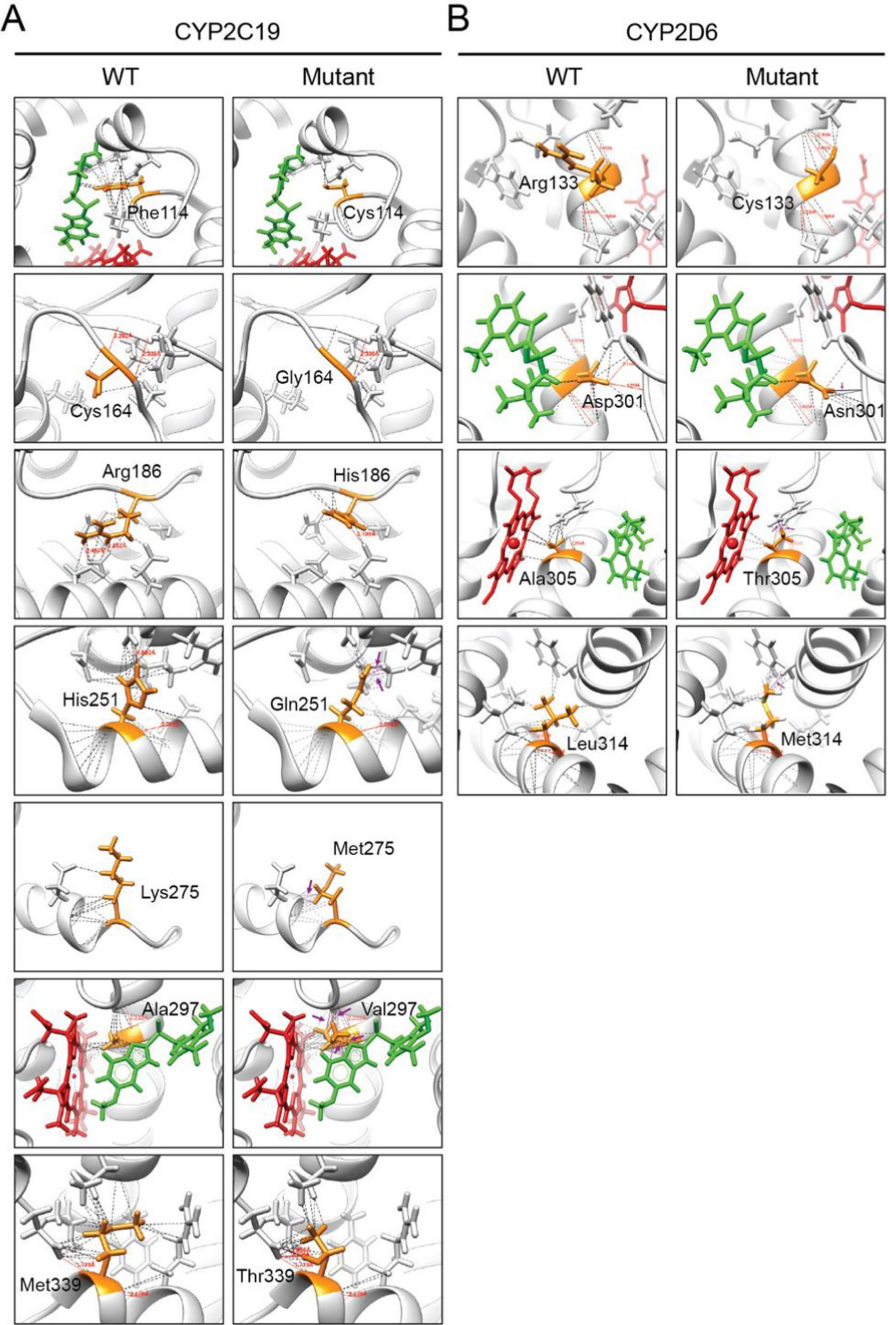
In silico modelling of substrates binding to CYP2C19.1 and CYP2D6.1 compared to variant enzymes. **A** Molecular docking shows that omeprazole (in green sticks) interacts with Phe114 and Ala297 (in orange, interactions presented as dashed pseudo-bonds), and forms unfavourable clashes upon Ala297Val mutation (presented as purple arrows and purple pseudo-bonds), whereas the docking site is distant from five other variant positions. **B** Only Asp301 is predicted to be in close contact with bufuralol (in green sticks). Clashes were observed in all CYP2D6 variant structures, except for Arg133Cys, suggesting that this substitution does not cause significant structural changes. Heme is shown in red sticks. Pseudo-bonds with distance labelled indicate hydrogen bonds formed between the hydrogen bond donor and acceptor

To investigate the diminished metabolic capabilities of the CYP2C19 and CYP2D6 variants, in silico modelling was conducted. The stereoscopic structures at each mutation site were analysed to assess the interactions between the mutated residues and their surrounding atoms (Fig. 2 and Table 3). Using the CYP2C19.1 template, structural analysis revealed that F114 and A297 are integral to the active site of omeprazole, corroborating previous findings [24].

**Table 3:** The differences in the number of interactions formed between the references CYP2C19.1 and CYP2D6.1 and the different variants residue and the neighboring residues/molecules

	Number of interactions on wild-type protein	Number of interactions on mutant protein	Differences in interactions	Number of unfavorable clashes formed
CYP2C19 variant				
F114C	74	8	− 66	0
C164G	13	9	− 4	0
R186H	23	13	− 10	0
H251Q	48	71	23	10
K275M	12	26	14	1
A297V	47	133	86	12
M339T	38	32	− 6	0
CYP2D6 variant				
R133C	14	19	5	0
D301N	32	44	12	1
A305T	20	39	19	3
L314M	27	40	13	1

The A297V substitution resulted in a significant increase in interactions with neighbouring residues, the heme iron-containing prosthetic group, and omeprazole. Among these interactions, 12 unfavourable clashes were identified, which likely caused residue 297 to be repelled from neighbouring residues and the substrate omeprazole (Fig. 2A, Supplementary Fig. 1). These structural conflicts likely disrupted the original conformation of the CYP2C19 active site, impairing omeprazole binding (Supplementary Fig. 2).

Similarly, the H251Q substitution introduced multiple collisions with a neighbouring helix, leading to a marked reduction in catalytic activity. The high number of collisions may have caused substantial conformational changes in the helices, producing a structure unfavourable for omeprazole binding.

In contrast to A297V, residue F114 interacts directly with omeprazole and neighboring residues (Fig. 2A) and thus stabilizes the binding of omeprazole to the active site. By replacing this phenylalanine to cysteine, this residue no longer interacts with omeprazole, and the original number of interactions between F114 and the neighboring residues is significantly reduced from 74 to 8 (Table 3). This decrease in interactions could destabilize the surrounding residue.

Which could change the orientation of the side chains of the neighboring residues and lower the number of interactions with omeprazole. The overall weaker interaction between C114 and omeprazole thus would be disadvantageous to the binding of omeprazole to the active site of this CYP2C19 variant (Supplementary Fig. 2).

For C164G and M339T, only minimal changes in the interactions were observed (Fig. 2A). However, the significantly lower catalytic activity of C164G compared to M339T suggests that C164 plays a crucial role in favoring omeprazole binding. Similarities of atomic interactions after amino acid replacement in residues 186 and 275 were noted (Fig. 2A). Nevertheless, R186 appears to play a more critical role in favoring omeprazole binding, as shown by the in vitro data, than the exchange for histidine.

For the four variants of CYP2D6, the exchange of amino acids only slightly altered the number of interactions with neighboring residues (Fig. 2B, Table 3). Clashes were predicted for D301N, A305T and L314M, indicating their influence on protein structure (Supplementary Fig. 1). Of note, both D301 and A305 are located in the active center of CYP2D6 [20]. It is therefore to be expected that amino acid exchange of both residues, respectively, impair the stability of substrate binding and corresponds well to the results from the in vitro experiments.

### Effects on access and egress channels

**Fig. 3:**
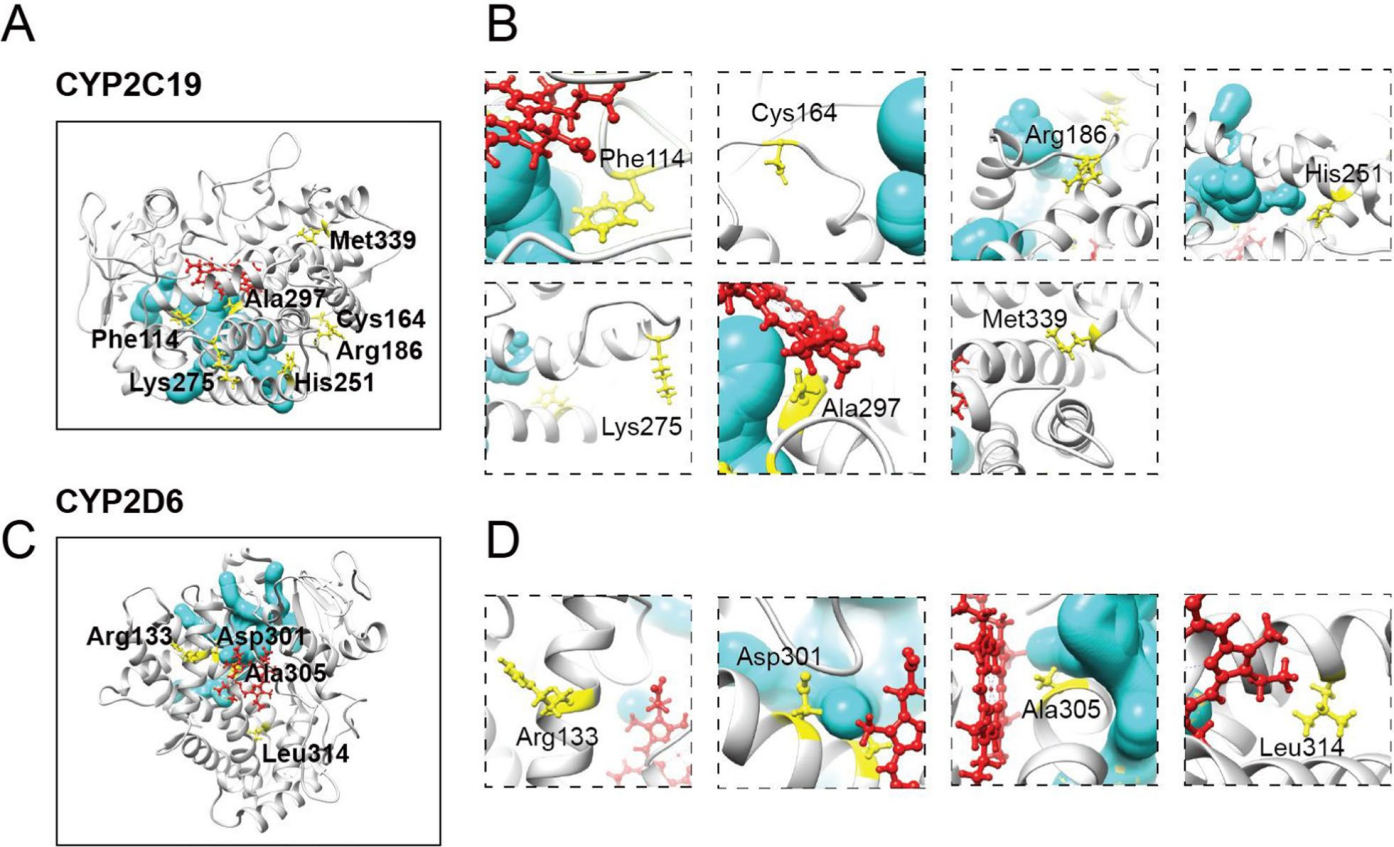
Access/egress channels identified in CYP2C19 and CYP2D6. **A**, **B** The predicted access and egress channel in CYP2C19 (in cyan) indicates that substrate binding is likely to be affected by mutations at Phe114, Arg186, His251, and Ala297 (in yellow sticks), due to their close proximity, but not by mutations at Cys164, Lys275, and Met399. **C**, **D** Similarly, access and egress of CYP2D6 substrate is likely to be affected by mutations at Asp301 and Ala305, but not by mutations at Arg133 and Leu314. Heme is shown in red sticks

Access and exit channels are pathways through which substrates and catalytic products of CYP2C19 and CYP2D6 must pass [22]. These channels provide important information about how substrates bind to the active center and how water and products exit. Since the mutation sites are located far away from the membrane, the influx and efflux channels were modelled and analyzed. Different CYP450s have different channels, and the entry and exit channels of CYP2C19 and CYP2D6 were modelled using MOLEOnline (Fig. 3). For CYP2C19, F114, R186, H251 and A297 are predicted to be near the entry and exit channels (Fig. 3A, B). The amino acid exchanges likely limit the entry of substrate and the exit of water molecules or metabolites. The entry and exit channels of CYP2D6 are also likely to be affected by the D301N and A305T exchanges, Fig. 3C, D).

## Discussion

The *CYP2C19* and *CYP2D6* genes are highly polymorphic, with several variants leading to the formation of enzymes with different metabolising abilities. In this study, we have thoroughly characterised in a proof-of-principle study how seven rare genetic variants in the *CYP2C19* gene and four genetic variants in the *CYP2D6* gene cause the formation of enzymes with altered catalytic activities compared to the reference variants CYP2C19.1 and CYP2D6.1. The CYP2C19 variants H251Q and A297V showed the lowest catalytic activities in accordance with both the ADME-optimised algorithm and the in vitro validation tests. However, there were discrepancies in the assessment of the catalytic activities of the other variants. In the in vitro assays, the CYP2C19 variants F114C and R186H retained approximately 20% of the normal catalytic activity, while the variant K275M had almost 40% of the normal catalytic activity of the enzyme. Conversely, the M339T variant showed the lowest reduction with 70% of the metabolising capabilities compared to CYP2C19.1. The predictive power of the ADME-optimised algorithm was highest for the most functionally different CYP2C19 variants, H251Q and A297V, and comparatively lower for variants with less decrease of activity such as K275M and M339T. 

In vitro analysis of the hydroxylation activity of bufuralol revealed that the CYP2D6 variant D301N had virtually no activity, although the prediction tool indicated 20–30%. Previous studies have identified D301 of CYP2D6 as a critical activity residue, with site-directed mutagenesis revealing intact substrate binding but a severe loss of catalytic activity [25]. This aspect is not taken into account in the ADME prediction tool, which explains the discrepancy between prediction and experimental results. For the CYP2D6 variants R133C and A305T, the prediction showed a lack of activity, which was close to the results of the in vitro analysis, which showed an activity of 5% and 10%, respectively. Similar to CYP2C19, the variant L314M, which had the highest catalytic activity (almost 60% compared to CYP2D6.1), showed the least success in prediction as the algorithm only predicted 0–10% activity.

While overall the in silico predictions aligned well with the results from in vitro experiments, the performance of these tools are far from perfect. The prediction tool used in this study has recently been improved by incorporating protein structural context provided by AlphaFold [26]. However, inferring variant function from structural information can be challenging since variants impairing enzymatic functions could be far from substrate binding sites as well as access and exit channels (Figs. 2 and 3). For example, our data showed that R133C is the second most deleterious variant in CYP2D6, resulting in the enzymatic function decreased to 4.6% (Table 2). However, this variant is neither in the previously well-defined CYP2D6 active site cavity [20], nor close to the channels predicted for substrate access and exit. These results suggested that there is still a long way to go before fully understanding the mechanisms of variant effect on metabolic enzyme functions. While machine learning-based computational methods provide appealing solutions, large-scale and high-quality variant function data, such as data generated from deep mutational scanning methods [27], are urgently required for model training, validation and testing. 

### Limitations and future directions

One limitation of this study is the use of only one CYP2C19 and one CYP2D6 substrate to determine catalytic activities. CYP enzymes exhibit high catalytic promiscuity, and CYP2C19 has various catalytic and inhibitory parameters. Substrate choice significantly influences CYP variant metabolizing activity, introducing complexity when optimizing the algorithm. Additionally, interpreting predicted results may require further investigation, as seemingly higher or lower predicted functional impacts may not accurately reflect the average metabolizing activity across all substrates for a given CYP enzyme.

While molecular docking was employed in this study to interrogate the effects of variants on substrate binding, this method is intrinsically limited in capturing the entire binding process and in interpreting the impact of variants located far from the active binding sites. In this context, molecular dynamics (MD) simulations could be useful to further elucidate the deleterious effects of the studied variants on substrate binding in CYP2C19 and CYP2D6.

Overall, these findings highlight the complexity of predicting enzyme activity based on genetic variants and underscore the importance of combining in silico modelling with in vitro validation for accurate functional predictions.

## Supplementary Information

Additional file 1: Figure S1. Highlighted clashes formed upon mutations in CYP2C19 and CYP2D6. Purple lines indicate clashes identified when conducting mutagenesis modeling in protein-drug complexes (corresponding to the six mutations in Fig. 2). The atomic distances at the sites of clashes (dashed lines) were quantified according to the method described in the Material and methods section

Additional file 2: Figure S2. Predicted substrate binding upon CYP2C19 and CYP2D6 mutations. Binding poses of drug substrates in wild-type (WT, green sticks) and mutant proteins were compared using both a superimposed overview (drug poses in mutants as wires) and separate visualizations.

Additional file 3: Table S1. DNA sequences of mutagenesis primers. The primer design was made with the QuikChange Primer design Program available om Agilent’s web site. Site of mutations are indicated with bold and underlined red letters. Table S2. Results obtained from the prediction tool ANNOVAR.

## Data Availability

No datasets were generated or analysed during the current study.
