# Supplementary figures and images for: Proof of principle concept for the analysis and functional prediction of rare genetic variants in the CYP2C19 and CYP2D6 genes
Source: Hum Genomics. 2025 May 28;19:62. doi: 10.1186/s40246-025-00765-2 (PMC12117788; doi:10.1186/s40246-025-00765-2)

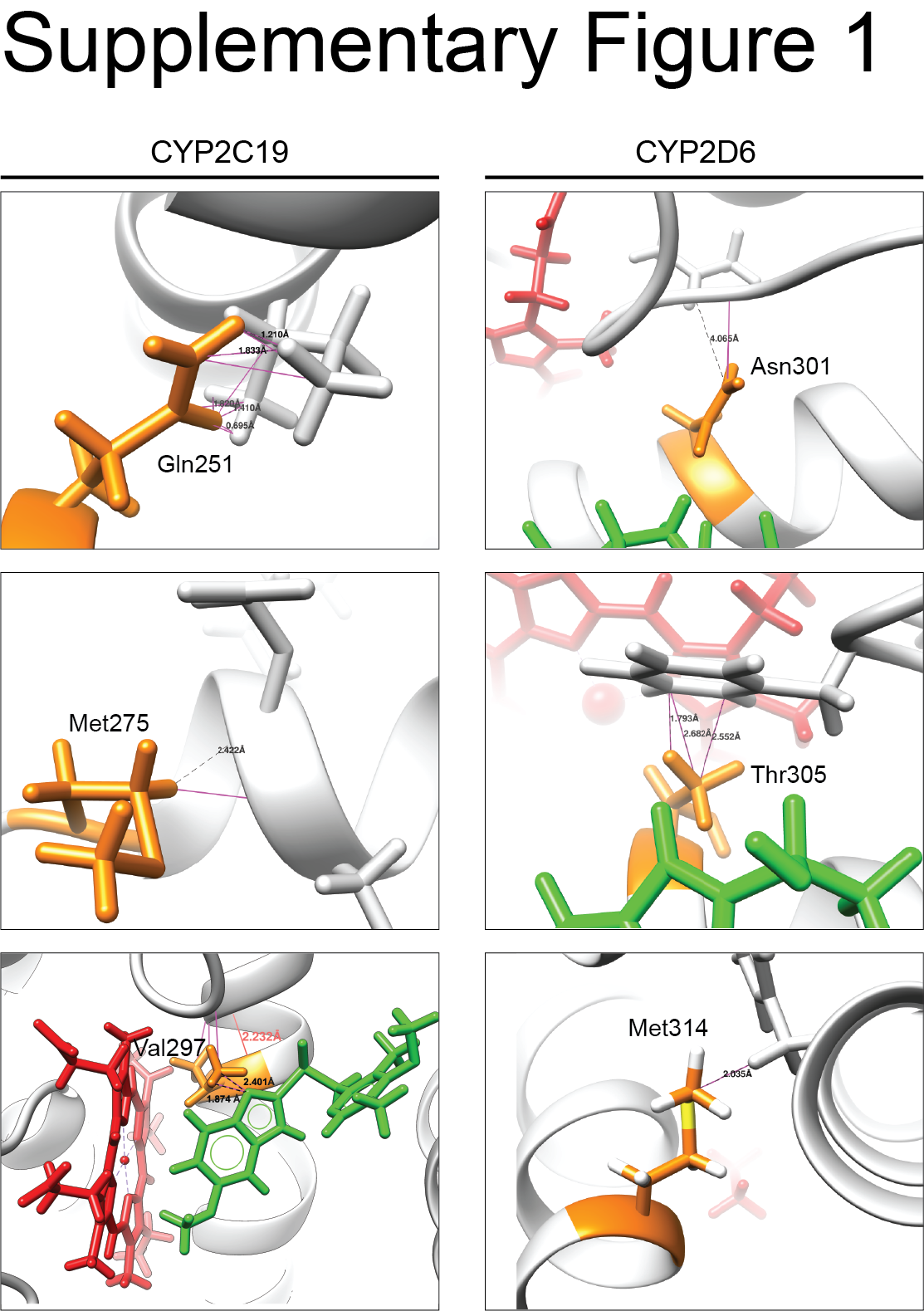

Supplement: Supplementary file 1 — Additional file 1: Figure S1. Highlighted clashes formed upon mutations in CYP2C19 and CYP2D6. Purple lines indicate clashes identified when conducting mutagenesis modeling in protein-drug complexes (corresponding to the six mutations in Fig. 2). The atomic distances at the sites of clashes (dashed lines) were quantified according to the method described in the Material and methods section [file 40246_2025_765_MOESM1_ESM.png]

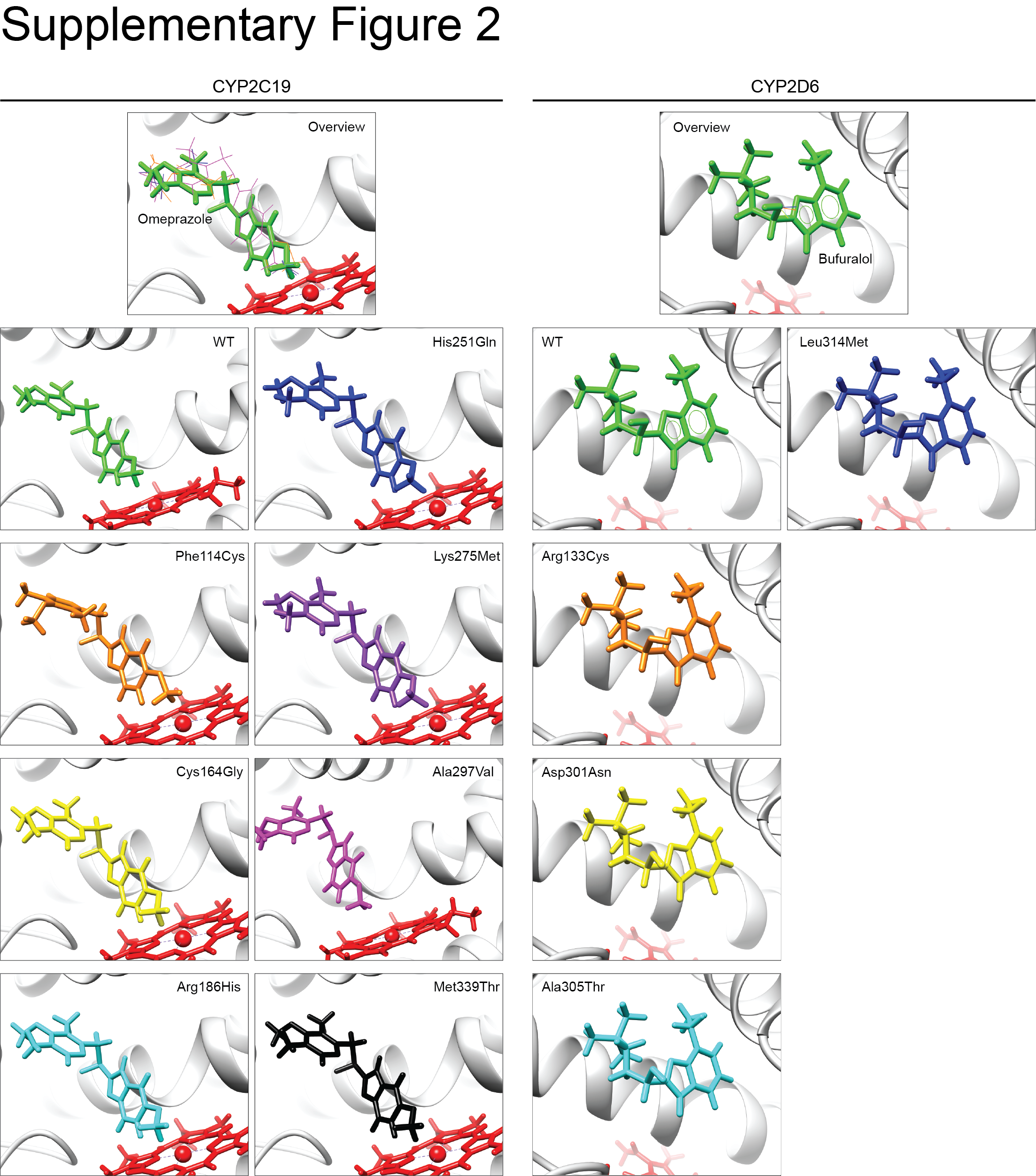

Supplement: Supplementary file 2 — Additional file 2: Figure S2. Predicted substrate binding upon CYP2C19 and CYP2D6 mutations. Binding poses of drug substrates in wild-type (WT, green sticks) and mutant proteins were compared using both a superimposed overview (drug poses in mutants as wires) and separate visualizations. [file 40246_2025_765_MOESM2_ESM.png]
