# Supplementary material for: Proof of principle concept for the analysis and functional prediction of rare genetic variants in the CYP2C19 and CYP2D6 genes
Source: Hum Genomics. 2025 May 28;19:62. doi: 10.1186/s40246-025-00765-2 (PMC12117788; doi:10.1186/s40246-025-00765-2)
Supplement: Supplementary file 3 — Additional file 3: Table S1. DNA sequences of mutagenesis primers. The primer design was made with the QuikChange Primer design Program available om Agilent’s web site. Site of mutations are indicated with bold and underlined red letters. Table S2. Results obtained from the prediction tool ANNOVAR. [file 40246_2025_765_MOESM3_ESM.docx]

**Supplementary Table 1.** DNA sequences of mutagenesis primers. The primer design was made with the QuikChange Primer design Program available om Agilent’s web site. Site of mutations are indicated with bold and underlined red letters.

|  | **Primer sequence** |
| --- | --- |
| **CYP2C19** |  |
| **P114C-F** | 5'-Ctaacagaggatttggaatcgttt**g**cagcaatggaaagag-3’ |
| **P114C-R** | 5'-ctctttccattgctg**c**aaacgattccaaatcctctgttag-3' |
| **C164G-F** | 5'-aaaccaaggcttcaccc**g**gtgatcccactttcatc-3' |
| **C164G-R** | 5'-gatgaaagtgggatcac**c**gggtgaagccttggttt-3' |
| **R186H-F** | 5'-ctgctccattattttccagaaac**a**tttcgattataaagatcagcaat-3' |
| **R186H-R** | 5'-attgctgatctttataatcgaaa**t**gtttctggaaaataatggagcag-3' |
| **H251Q-F** | 5'-tttggagaaagtaaaagaaca**g**caagaatcgatggacatcaac-3' |
| **H251Q-R** | 5'-gttgatgtccatcgattcttg**c**tgttcttttactttctccaaa-3' |
| **K275M-F** | 5'-cttcctgatcaaaatggagaaggaaa**t**gcaaaaccaacagt-3' |
| **K275M-R** | 5'-actgttggttttgc**a**tttccttctccattttgatcaggaag-3' |
| **A297V-F** | 5'-agctgacttacttggag**t**tgggacagagacaacaa-3' |
| **A297V-R** | 5'-ttgttgtctctgtccca**a**ctccaagtaagtcagct-3' |
| **M339T-F** | 5'-cggagcccctgca**c**gcaggacaggg-3' |
| **M339T-R** | 5'-ccctgtcctgc**g**tgcaggggctccg-3' |
| **CYP2D6** |  |
| **R133C-F** | 5’- GCGCGAGCAGAGG**T**GCTTCTCCGTGTC-3’ |
| **R133C-R** | 5’- GACACGGAGAAGCACCTCTGCTCGCGC-3’ |
| **D301N-F** | 5’- CTGCGCATAGTGGTGGCT**A**ACCTGTTCTCTG-3’ |
| **D301N-R** | 5’- CAGAGAACAGGT**T**AGCCACCACTATGCGCAG-3’ |
| **A305T-F** | 5’-GGTGGCTGACCTGTTCTCT**A**CCGGGATGGT-3´ |
| **A305T-R** | 5’-ACCATCCCGG**T**AGAGAACAGGTCAGCCACC-3´ |
| **L314M-F** | 5’- CCTCGACCACG**A**TGGCCTGGGGC-3’ |
| **L314M-R** | 5’- GCCCCAGGCCA**T**CGTGGTCGAGG-3’ |

**Supplementary Table 2**

Results obtained from the prediction tool ANNOVAR.

| **Amino acid exchange** |  | | | | | **Comparing to ADME -optimized threshold**  **(according to Zhou 2019)** | | | | |  |  |
| --- | --- | --- | --- | --- | --- | --- | --- | --- | --- | --- | --- | --- |
|  | **LRT_**  **score** | **Muta-tion**  **Asses-sor_**  **score** | **PRO-VEAN_**  **score** | **VEST3**  **score** | **CADD**  **phred** | **LRT** | **Muta-tion Asses-sor** | **PRO-VEAN** | **VEST3** | **CADD**  **phred** | **ADME**  **score** | **ADME**  **predic-tion** |
| **CYP2C19** | | | | | | | | | | | | |
| F114C | 0.066 | 3.055 | -6.71 | 0.711 | 25.1 | 0 | 1 | 1 | 1 | 1 | 0.8 | D |
| C164G | 0.001 | 1.555 | -10.59 | 0.649 | 22.4 | 1 | 0 | 1 | 1 | 1 | 0.8 | D |
| R186H | 0 | 2.95 | -3.22 | 0.581 | 18.65 | 1 | 1 | 0 | 1 | 0 | 0.6 | D |
| H251Q | 0 | 3.01 | -6.98 | 0.309 | 23.4 | 1 | 1 | 1 | 0 | 1 | 0.8 | D |
| K275M | 0 | 3.01 | -4.6 | 0.17 | 23.1 | 1 | 1 | 1 | 0 | 1 | 0.8 | D |
| A297V | 0 | 3.855 | -3.64 | 0.817 | 23.1 | 1 | 1 | 1 | 1 | 1 | 1 | D |
| M339T | 0.001 | 1.745 | -5.36 | 0.324 | 21.2 | 1 | 0 | 1 | 0 | 1 | 0.6 | D |
| **CYP2D6** | | | | | | | | | | | | |
| R133C | 0.001 | 4.15 | -6.54 | 0.755 | 26.4 | 1 | 1 | 1 | 1 | 1 | 1 | D |
| D301N | 0 | 1.995 | -4.48 | 0.373 | 20.5 | 1 | 0 | 1 | 0 | 1 | 0.6 | D |
| A305T | 0 | 3.77 | -3.7 | 0.917 | 24.8 | 1 | 1 | 1 | 1 | 1 | 1 | D |
| L314M | 0 | 2.515 | -1.61 | 0.538 | 23.3 | 1 | 1 | 0 | 1 | 1 | 0.8 | D |

**Supplementary Figure 1. Highlighted clashes formed upon mutations in CYP2C19 and CYP2D6.** Purple lines indicate clashes identified when conducting mutagenesis modeling in protein-drug complexes (corresponding to the six mutations in Figure 2). The atomic distances at the sites of clashes (dashed lines) were quantified according to the method described in the Material and methods section.

**Supplementary Figure 2. Predicted substrate binding upon CYP2C19 and CYP2D6 mutations.** Binding poses of drug substrates in wild-type (WT, green sticks) and mutant proteins were compared using both a superimposed overview (drug poses in mutants as wires) and separate visualizations.
